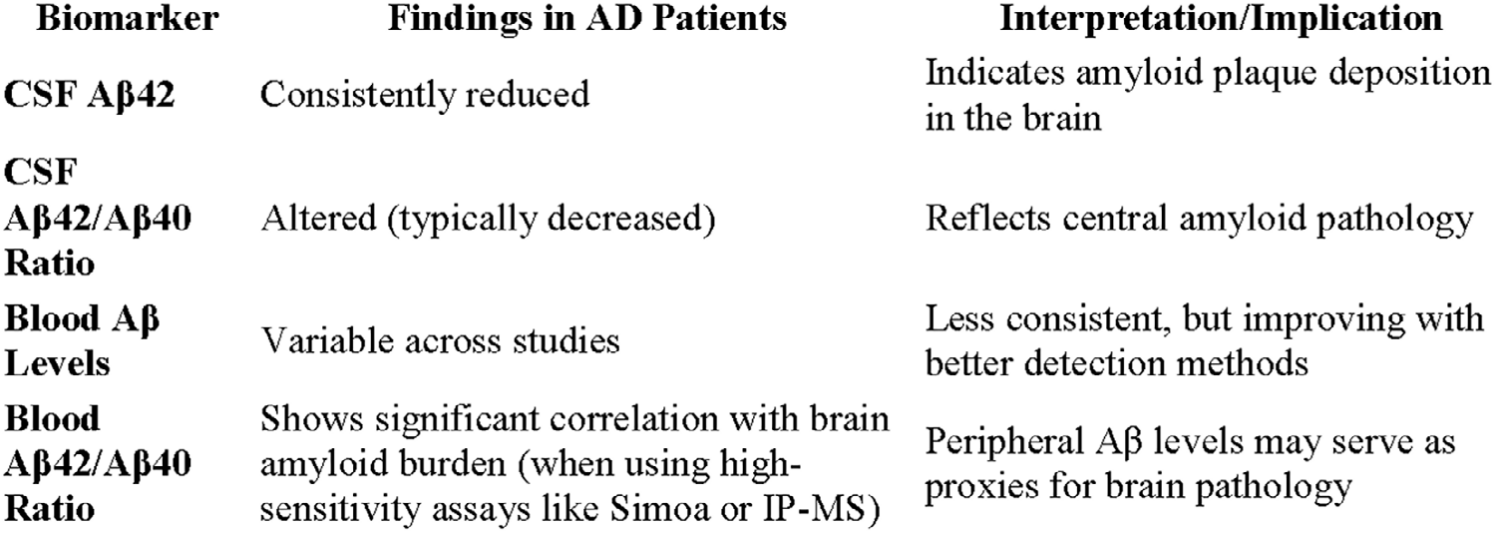# The Role of Amyloid‐Beta in Blood and Cerebrospinal Fluid: Implications for Alzheimer’s Disease Diagnosis and Monitoring

**DOI:** 10.1002/alz70861_108285

**Published:** 2025-12-23

**Authors:** Chanikarn Sonpee, Kanthita Worachotsueptrakun, Chanida Ruchisrisarod, Watayuth Luechaipanit, Pasin Hemachudha, Abhinbhen Wasontiwong Saraya

**Affiliations:** ^1^ Kingchulalongkorn Memorial Hospital, Pathumwan, Bangkok Thailand; ^2^ King Chulalongkorn Memorial Hospital, Pathumwan, Bangkok Thailand; ^3^ Thai Red Cross Emerging Infectious Diseases Health Science Centre, King Chulalongkorn Memorial Hospital, Bangkok Thailand; ^4^ Faculty of Medicine, Chulalongkorn University, Bangkok Thailand; ^5^ Chula Neuroscience Centre, Bangkok, Bangkok Thailand; ^6^ Division of Neurology, Department of Medicine, Faculty of Medicine, Chulalongkorn University, Bangkok Thailand

## Abstract

**Background:**

Alzheimer’s disease (AD) is a progressive neurodegenerative disorder characterized by cognitive decline and memory impairment. One of the hallmark pathological features of AD is the accumulation of amyloid‐beta (Aβ) peptides in the brain. Traditionally, cerebrospinal fluid (CSF) biomarkers, particularly Aβ42, have been central to the diagnosis and monitoring of AD. However, growing evidence supports the utility of blood‐based Aβ measurements as less invasive and more accessible biomarkers.

**Methods:**

This review synthesizes findings from recent clinical studies and meta‐analyses that evaluate Aβ concentrations in both CSF and blood. Studies comparing Aβ42, Aβ40, and their ratios in AD patients, mild cognitive impairment (MCI) individuals, and healthy controls were examined. Correlations between fluid biomarkers and imaging or cognitive outcomes were also assessed.

**Results:**

A consistent reduction in CSF Aβ42 and altered Aβ42/Aβ40 ratios were observed in AD patients compared to controls, reflecting amyloid plaque deposition in the brain. Blood Aβ levels showed more variability across studies but demonstrated promise, particularly when analyzed with high‐sensitivity platforms such as immunoprecipitation‐mass spectrometry or Simoa (Single Molecule Array). Significant correlations between plasma Aβ42/Aβ40 ratios and brain amyloid burden were reported, suggesting peripheral Aβ may reflect central pathology.

**Conclusion:**

Aβ levels in CSF remain a gold standard for early AD detection and disease monitoring. However, advancements in blood‐based biomarker assays offer a less invasive and potentially scalable approach for population‐level screening and longitudinal monitoring. Continued refinement of analytical techniques and standardization across studies are essential to establish the clinical utility of blood‐based Aβ biomarkers in AD.